# Characterization of the Genetic Architecture for Fusarium Head Blight Resistance in Durum Wheat: The Complex Association of Resistance, Flowering Time, and Height Genes

**DOI:** 10.3389/fpls.2020.592064

**Published:** 2020-12-23

**Authors:** Yuefeng Ruan, Wentao Zhang, Ron E. Knox, Samia Berraies, Heather L. Campbell, Raja Ragupathy, Kerry Boyle, Brittany Polley, Maria Antonia Henriquez, Andrew Burt, Santosh Kumar, Richard D. Cuthbert, Pierre R. Fobert, Hermann Buerstmayr, Ron M. DePauw

**Affiliations:** ^1^Swift Current Research and Development Centre, Agriculture and Agri-Food Canada, Swift Current, SK, Canada; ^2^Aquatic and Crop Resource Development Research Centre, National Research Council of Canada, Saskatoon, SK, Canada; ^3^Lethbridge Research and Development Centre, Agriculture and Agri-Food Canada, Lethbridge, AB, Canada; ^4^Morden Research and Development Centre, Agriculture and Agri-Food Canada, Morden, MB, Canada; ^5^Ottawa Research and Development Centre, Agriculture and Agri-Food Canada, Ottawa, ON, Canada; ^6^Brandon Research and Development Centre, Agriculture and Agri-Food Canada, Brandon, MB, Canada; ^7^Aquatic and Crop Resource Development Research Centre, National Research Council of Canada, Ottawa, ON, Canada; ^8^University of Natural Resources and Life Sciences, Vienna, Austria; ^9^Advancing Wheat Technology, Swift Current, SK, Canada; ^10^Retired from Swift Current Research and Development Centre, Agriculture and Agri-Food Canada, Swift Current, SK, Canada

**Keywords:** resistance, QTL, GWAS, Fusarium head blight, durum

## Abstract

Durum wheat is an economically important crop for Canadian farmers. Fusarium head blight (FHB) is one of the most destructive diseases that threatens durum production in Canada. FHB reduces yield and end-use quality and most commonly contaminates the grain with the fungal mycotoxin deoxynivalenol, also known as DON. Serious outbreaks of FHB can occur in durum wheat in Canada, and combining genetic resistance with fungicide application is a cost effective approach to control this disease. However, there is limited variation for genetic resistance to FHB in elite Canadian durum cultivars. To explore and identify useful genetic FHB resistance variation for the improvement of Canadian durum wheat, we assembled an association mapping (AM) panel of diverse durum germplasms and performed genome wide association analysis (GWAS). Thirty-one quantitative trait loci (QTL) across all 14 chromosomes were significantly associated with FHB resistance. On 3BS, a stable QTL with a larger effect for resistance was located close to the centromere of 3BS. Three haplotypes of *Fhb1* QTL were identified, with an emmer wheat haplotype contributing to disease susceptibility. The large number of QTL identified here can provide a rich resource to improve FHB resistance in commercially grown durum wheat. Among the 31 QTL most were associated with plant height and/or flower time. QTL 1A.1, 1A.2, 3B.2, 5A.1, 6A.1, 7A.3 were associated with FHB resistance and not associated or only weakly associated with flowering time nor plant height. These QTL have features that would make them good targets for FHB resistance breeding.

## Introduction

Fusarium head blight (FHB), also known as scab and mainly caused by *Fusarium graminearum* Schwabe [teleomorph: *Gibberella zeae* (Schwein.) Petch] (Bai and Shaner, 1994; McMullen et al., 1997), is a devastating fungal disease of small-grain cereals including durum and common wheat and barley, resulting in severe yield and quality losses (Gilbert and Tekauz, 2000; McMullen et al., 2012). Moreover, as food for humans and feed for animals, FHB infected grain also creates health risks due to contamination with mycotoxins. This is a particular concern for durum wheat, as its main purpose is for human consumption (Bai and Shaner, 2004; Zhao et al., 2018; Haile et al., 2019; He et al., 2019). Canada is the largest producer and exporter of durum wheat supplying more than a half of the world’s total exported durum (International Grains Council, 2020). Since the early 1990s, FHB has become the major disease threatening durum production in Canada and has caused major economic losses for producers (Gilbert and Tekauz, 2000). In 2016, a severe FHB epidemic caused 65% of the common wheat and 36% of the durum wheat to be downgraded in Saskatchewan, Canada, with an estimated economic loss of $1 billion (Canadian Grain Commission, 2017). It is therefore a priority to develop durum wheat with desirable FHB resistance to protect it from losses.

Currently, the combination of agronomic and chemical control along with genetic resistance is the most effective means to manage FHB (Gilbert and Haber, 2013; Prat et al., 2014). Genetic resistance is preferred due to its lower cost, higher efficacy, and environmental benefit (Prat et al., 2014). Genetic resistance to FHB in wheat is quantitative in expression due to control by multiple minor genes. FHB resistance is also significantly affected by environment (Bai and Shaner, 2004; Buerstmayr et al., 2009, 2019), thus having lower to moderate heritability (Van Sanford et al., 2001). Therefore, when visual assessment of FHB is performed in the field, lines must be tested in multiple independent environments with intensive phenotyping to reliably identify QTL for resistance.

Developmental traits including flower time, plant height, spike morphology, and anther extrusion/or retention are often reported for their relationship with FHB resistance (Mesterhazy, 1995; Gervais et al., 2003; Srinivasachary et al., 2009; Skinnes et al., 2010; Lv et al., 2014; Buerstmayr and Buerstmayr, 2016). Plant height and disease resistance mostly show a significantly negative correlation (Mesterhazy, 1995; Srinivasachary et al., 2009; Buerstmayr and Buerstmayr, 2016). Pleiotropic effects, tightly linked genes and disease escape have all been hypothesized as feasible mechanisms for resistance related to these developmental traits.

Fusarium head blight resistance can be categorized into three main types or components: (1) type I – resistance to initial infection measured by the incidence of disease in the presence of natural or augmented artificial inoculum (e.g., spray inoculation); (2) type II – resistance to fungal spread measured by the severity of disease; and (3) type III – resistance to the accumulation of the toxin deoxynivalenol (DON) in infected spikes (Miller et al., 1985; Mesterhazy, 1995; Bai and Shaner, 2004). Till now, more than 556 QTL contributing to FHB resistance have been identified on all 21 chromosomes of hexaploid wheat (Buerstmayr et al., 2009; Liu et al., 2009; Löffler et al., 2009; Venske et al., 2019). These QTL can be refined largely into 56 clusters by meta-QTL analysis (Venske et al., 2019). In spite of the relatively large number of identified QTL for FHB, only three QTL, *Fhb1* on chromosome arm 3BS (Anderson et al., 2001; Liu et al., 2006), *Qfhs.ifa-5A* on 5AS (*Fhb5*) (Buerstmayr et al., 2002; Somers et al., 2003; Steiner et al., 2019a) and *Fhb2* on 6BS (Anderson et al., 2001; Cuthbert et al., 2007) have been validated. All of these resistance loci originate from the Chinese cultivar Sumai 3, which displays among the highest levels of FHB resistance observed (Buerstmayr et al., 2009). *Fhb1* is the best validated, and most frequently studied and deployed resistance QTL (Buerstmayr et al., 2019). It is currently the only resistance QTL confirmed to be present in several new FHB North American and European varieties with strong resistance (Hao et al., 2019). *Fhb1* is reported primarily as conferring strong Type II resistance, and accounting for 20–60% of phenotypic variation in breeding populations (Miedaner and Korzun, 2012). *Fhb1* was recently claimed to be cloned by three research groups as two different candidate genes (Rawat et al., 2016; Li et al., 2019; Su et al., 2019) with conflicting interpretations, leaving room for independent validation.

Compared to the large amount of genetic variation for FHB resistance reported in common wheat, durum wheat has limited sources of resistance (Oliver et al., 2008; Prat et al., 2014; Steiner et al., 2019b). Tetraploid sources of FHB resistance that have been identified include the Canadian durum cultivar Strongfield (Somers et al., 2006), experimental line DT696 (Sari et al., 2018), *T. carthlicum* (Somers et al., 2006; Oliver et al., 2008; Sari et al., 2018), *T. dicoccoides* (Ruan et al., 2012), *T. dicoccum* (Buerstmayr et al., 2012; Zhang et al., 2014), and Tunisian durum landraces (Ghavami et al., 2011; Huhn et al., 2012). Among these findings, the most stable and consistent QTL were identified on chromosomes 2B, 3A, 3B, and 5A (Prat et al., 2014; Haile et al., 2019).

As hexaploid wheat has significantly more sources of FHB resistance, introgression of resistance from hexaploid into durum wheat is one possible way to expand the durum resistance gene pool. Previous attempts to introgress FHB resistance from Sumai 3 into durum were largely unsuccessful (Prat et al., 2017). However, several recent successes have been reported with *Fhb1* from Sumai 3 (Giancaspro et al., 2016; Prat et al., 2017) as well as a non-Sumai 3-related FHB resistance sources (Chu et al., 2011; Zhao et al., 2018). Despite these partial successes, no commercial durum cultivars with QTL from these non-adapted sources have been released due to the lengthy breeding process, linkage drag or suppression of resistance in durum backgrounds. Because of these challenges, utilizing the FHB resistance already present in durum cultivars is gaining favor as a promising approach to bring durum wheat cultivars with improved resistance to market more quickly. Durum cultivars with an improved level of FHB resistance have been developed and released by the North Dakota durum breeding program using this strategy (Zhang et al., 2014). With the same approach, recent durum cultivars, including Brigade (Clarke et al., 2009) and Transcend (Singh et al., 2012) with a better level of FHB resistance have also been successfully developed and released by Canadian durum breeding programs selecting for reduced symptoms in FHB nurseries. Regardless of this initial success, there is still a need to know and identify additional native sources of resistance as well as more exotic sources. Understanding the association of FHB resistance with developmental traits, flowering time and plant height is also important for recommending which resistance loci may be most relevant to a particular breeding program.

Genome wide association studies (GWAS) are a promising way to detect FHB resistance QTL present in diverse genetic sources. Only a few GWAS have been conducted on FHB resistance, including winter wheat (Wang et al., 2017), elite Chinese wheat (Zhu et al., 2020), durum breeding panels (Steiner et al., 2019b) and type II FHB resistance durum diversity panels (Ghavami et al., 2011). In this study, we aimed to use GWAS to explore FHB resistance of domestic durum cultivars and breeding material as well as exotic sources of resistance, including Sumai 3 and emmer wheat introgression lines. With GWAS in multiple environments, we aimed to: 1) explore and characterize FHB resistance QTL in durum wheat from the domestic as well as exotic sources, and 2) identify resistance QTL that colocalize with flowering time and plant height.

## Materials and Methods

### Plant Materials

In total, 186 diverse durum wheat (*Triticum turgidum* L. ssp. *Durum* (Desf.) Husn.) lines were selected to constitute a durum association mapping (AM) panel targeted to improve FHB resistance in durum wheat. This panel was primarily composed of durum from Canada, including elite Canadian cultivars, advanced breeding lines, recently developed germplasm from Canadian breeding programs and from research projects (Supplementary Table 1). Experimental durum lines representing exotic FHB resistance and germplasm from global collections made up the remainder of the AM panel (Supplementary Table 1).

### Phenotyping

Lines of the durum AM panel were evaluated for FHB infection in Morden and Brandon, MB, Canada in 2015 to 2017 with artificial inoculation and Indian Head, SK, Canada in 2015 and 2016 with natural infection. At both Morden and Brandon, FHB nurseries, corn spawn inoculum of *Fusarium graminearum* was used. Corn spawn consisted of grains that were inoculated with a mixture of two *F*. *graminearum* isolates, a 3-acetyl-deoxynivalenol (3ADON, M9-07-01) and a 15-acetyl-deoxynivalenol (15ADON, M1-07-02) isolate, after which colonized kernels were air dried. In Morden, approximately 2–3 weeks prior to heading, the corn spawn inoculum was spread at 8 g per single meter row with two applications at weekly intervals. Plots were irrigated three times per week using Cadman Irrigation travelers with Briggs booms. At Brandon, the corn spawn inoculum was applied between the rows at a rate of 40 g/m 6 weeks after planting, with a second application performed at the same rate 2 weeks after the first. Plots were irrigated three times per week with a mist irrigation system to create favorable conditions for *F. graminearum* infection. In Indian Head, FHB was achieved solely by natural disease infection. FHB incidence (INC, percentage of spikes showing symptoms) and severity (SEV, average percentage of spike with visual symptoms of infection) were estimated with visual assessment. FHB index (IND) was calculated with the formula: (INC × SEV)/100. Plant height (HT) and days to anthesis (DTA) were also recorded for Morden plots.

### Genotyping

Genomic DNA of the durum AM panel was extracted from freeze-dried fresh leaf tissue of seedlings with a CTAB based protocol carried out on an automated AutoGen DNA isolation system (AutoGen, Holliston, MA). DNA was quantified with a Quant-iT^TM^ PicoGreen^®^ dsDNA Assay Kit (Thermo Fisher Scientific Inc., Bartlesville, OK, United States) and diluted to 50 ng/μL for SNP array genotyping. Genotyping of DNA was performed with the Illumina iSelect 90K SNP array (Wang et al., 2014) according to the manufacturer protocol (Illumina). SNP arrays were scanned with an Illumina HiScan. Raw intensity files from the HiScan were imported into GenomeStudio Version 2013 (polyploid clustering module v1.0.0, Illumina). SNP calling was performed with the method described by Wang et al. (2014) with 3 cluster steps of the cluster algorithm DBSCAN then OPTICS. All SNPs were subsequently visually checked, and incorrectly clustered SNPs or SNPs with more than 4 clusters were manually removed. Finally, SNPs with minor allele frequency (MAF) below 0.05, and missing genotypes higher than 15% were filtered out. This resulted in a total of 6900 high quality polymorphic SNPs of which 5933 markers were anchored to the wheat consensus map (Wang et al., 2014) for the downstream genome wide association analysis.

### Statistical Analysis

Statistical analysis was performed with R 3.4.2 (R Core Team, 2017) with the lme4 package (Bates et al., 2015). Phenotypic traits from each disease nursery site across multiple years were fitted with the linear mixed model (Bates et al., 2015). The model is implemented as: *P*_*iy*_ = μ + G_*i*_ + E_*y*_ + (G_*i*_XE_*y*_) + E_*iy*_, where, P_*iy*_ are the values of the tested phenotypic trait, μ is the population mean, G_*i*_ is the effect of genotypes, E_*y*_ is the effect of environments (here, by Year), E_*iy*_ is the residual, where i is the genotype, y is the year. The restricted maximum likelihood (REML) method within lme4 (Bates et al., 2015) was used to estimate the variance components of each trait. The broad sense heritability (*H*^2^) was estimated with the equation $H^{2}=\frac{\delta_{G}^{2}}{\delta_{G}^{2}+\frac{\delta_{GXE}^{2}}{y}+\frac{\delta_{e}^{2}}{p}}$ across multiple years in each disease nursery site, where: $\delta_{G}^{2}$ is the genotypic variance, $\delta_{GXE}^{2}$ is the variance of interaction between genotype and year, $\delta_{e}^{2}$ is the error variance, y is the number years, and p is the total number of replications in all tested years. The least squares means were used for trait correlation and association mapping analysis. The correlation coefficients of disease response, plant height and days to anthesis across multiple years and multiple sites were calculated with the Pearson correlation test and visualized with the R package “corrplot” (Wei and Simko, 2017).

### Linkage Disequilibrium, Population Structure, and Kinship Analysis

Linkage disequilibrium (LD) was estimated by correlation coefficient analysis and used the squared correlation coefficients (*r*^2^) for all 5933 anchored SNP markers implemented in Tassel v.5.5.0 (Bradbury et al., 2007). The *r*^2^ values of unlinked genetic markers (defined as genetic distance > 30 cM) were square-root transformed into a normal distribution. The baseline (or critical) *r*^2^ value, a value that suggested LD was likely caused by genetic linkage, was determined by taking the 95% percentile of this distribution (Breseghello and Sorrells, 2006). The scatter plot of *r*^2^ versus genetic distance (cM) was fitted using a non-linear model described by Remington et al. (2001) that was implemented in software PopLDdecay (Zhang et al., 2019).

Population structure of the durum AM panel was determined with STRUCTURE v2.3.4 (Pritchard et al., 2000) with a pruned SNP marker dataset that was generated with the LD (linkage disequilibrium)-based pruning approach implemented in the software PLINK (Purcell et al., 2007). A total of 2306 pruned markers with LD (*r*^2^) ≤ 0.2 were used for population structure analysis. STRUCTURE analysis was performed with a 50000 burn-in length and 100000 Markov chain Monte Carlo (MCMC) iterations from *K* = 2 to *K* = 12 (*K*, specialized clusters of the AM panel). Fifteen independent STRUCTURE runs were conducted for each specialized *K*. The optimal cluster (*K*) was determined by the ΔK method (Evanno et al., 2005), implemented in the software Structure Harvester (Earl and vonHoldt, 2012). Independent runs of the optimal K were summarized using CLUMMP v1.1.2 software (Jakobsson and Rosenberg, 2007). The CLUMMP generated Q matrix was used to graph the population structure using Structure Plot software (Ramasamy et al., 2014) and perform downstream GWAS analysis. A phylogenetic tree was built with the neighbor-joining (NJ) method in MEGA6 (Tamura et al., 2013) and visualized with Figtree v1.4.4^1^. Principal component analysis (PCA) was performed with R package Genome Association and Prediction Integrated Tools (GAPIT) (Lipka et al., 2012; Tang et al., 2016).

### Genome-Wide Association Study

Association mapping was performed on the durum association panel using the phenotypic data collected from the multiple nurseries in multiple years, including HT, DTA, INC, SEV and IND. Association mapping was performed using 5933 mapped SNPs that had a MAF > 0.05 using both Tassel v5.5.50 (Bradbury et al., 2007) and GAPIT (Lipka et al., 2012; Tang et al., 2016). Different association models were tested in both software packages, and QQ-plots generated from all the models were compared to select the model that best controls false positives and negatives. All the data presented here were generated in TASSEL using a mixed linear model (MLM) incorporating the STRUCTURE (Q) matrix as a fixed factor and the kinship (K) matrix as a random factor (Q + K MLM). To be considered a QTL in this dataset, we selected SNPs that were significant (*p* < 0.05, marker-wise) in at least four of the tested environments for FHB resistance or two for plant height and flower time, and with at least one environment with a highly significant response (*p* < 0.001). Significant SNPs on the same linkage group were grouped into a QTL region if markers were linked with LD > 0.2.

## Results

### Population Structure and Linkage Disequilibrium (LD) Analysis

**FIGURE 1:**
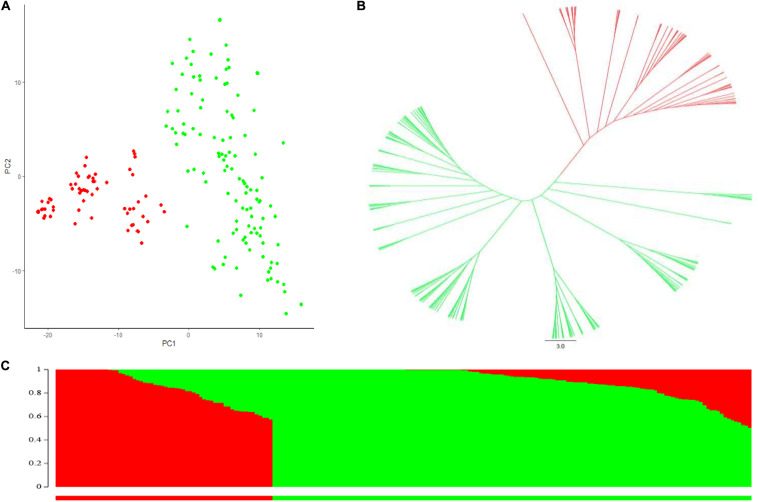
Population structure analysis of the durum association mapping (AM panel). **(A)** Principal component analysis (PCA). **(B)** Phylogenetic tree constructed with Neighbor Joining (NJ) method, green color and red color represented subpopulations 1 and 2 inferred from Structure analysis. **(C)** Population structure analysis with *K* = 2 of the AM panel. Green color, subpopulation 1 and red color subpopulation 2.

STRUCTURE analysis, principal component analysis (PCA) and NJ-phylogenetic tree analysis were all used to determine clustering of lines within the durum AM panel, and two subpopulations were consistently indicated, as shown by different colors in Figure 1. Subpopulation 1 (shown as green in Figure 1 and Supplementary Table 1) contained 124 lines, and consisted of a large proportion of Canadian cultivars and inbred lines including the older cultivar Kyle, more recent cultivars Strongfield and currently most popular cultivars as Brigade, Transcend and CDC Credence. Subpopulation 2 included 62 lines (shown in red in Figure 1 and Supplementary Table 1), consisting of the founder landrace Pelissier and the majority of lines from Austria. All of the inbreeding lines derived from introgression of FHB resistance genes from Sumai 3 into European durum wheat cultivars were contained in subpopulation 2, as were the majority of *T. dicoccoides* introgression lines. The baseline critical threshold *r*^2^ value of LD was identified as 0.2, corresponding to a genetic distance around 3.0 cM from the whole genome analysis (Supplementary Figure 1).

### Phenotypic Analysis

**FIGURE 2:**
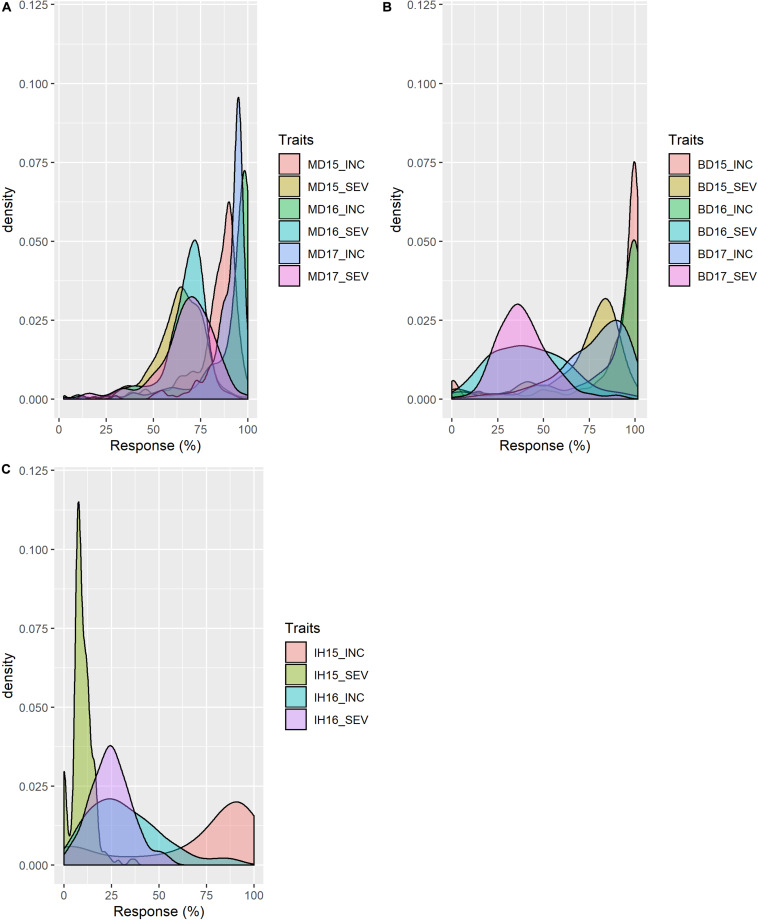
Distribution of FHB resistance of the durum association mapping panel (AM) in field trials at **(A)** Morden, MB; **(B)** Brandon, MB; and **(C)** Indian Head, SK. INC: incidence (%), percentage of spikes showing symptoms; SEV: severity (%), percentage of spike area infected. 15, 16 and 17: years 2015, 2016, and 2017.

**FIGURE 3:**
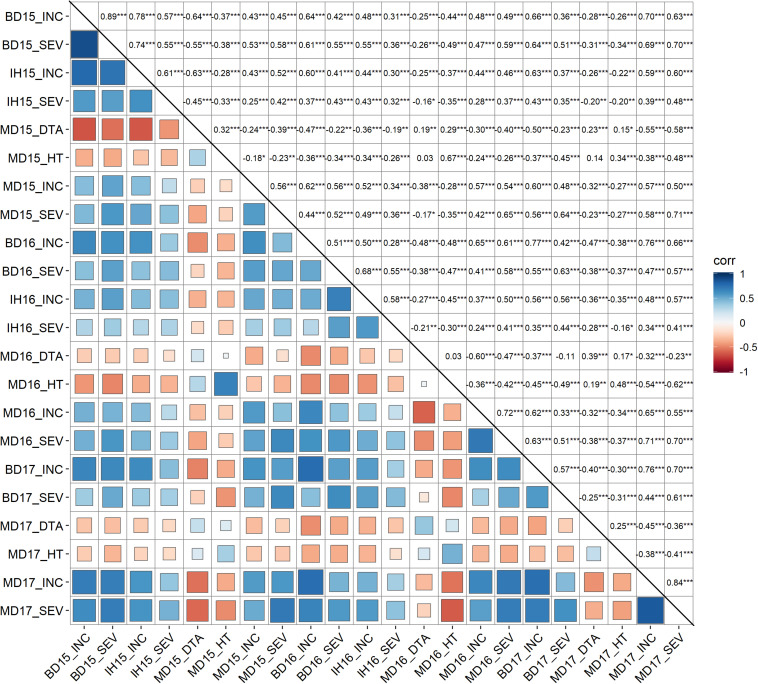
Pearson correlation analysis of fusarium head blight resistance related traits. INC, Incidence (%), FHB incidence, percentage of spikes showing symptoms; SEV, severity (%), percentage of spike area infected; HT, plant height (cM); and DTA, day to anthesis. MD, Morden, MB; BD, Brandon, MB; IH, Indian Head, SK; 15, 16, and 17, field trials in year 2015, 2016 and 2017. Correlation coefficients were shown in upper triangle. Levels of significance claimed at *P < 0.05; **p < 0.005, ***p < 0.0001.

Mean values (across years) of FHB INC, SEV, IND, DTA and HT of lines from the durum AM panel at Brandon, Morden, and Indian Head, were summarized in Supplementary Table 1. Across environments, FHB INC tended to be higher than SEV (Figure 2) which is reflected in the overall means (Table 1). The lowest INC was observed at Indian Head in 2016, the location with the lowest severities in both 2015 and 2016. Moderate SEV were observed at Brandon in 2016 and 2017. Generally, a large differential in FHB INC and SEV was observed as indicated by the range for each environment in Table 1, except Indian Head where the maximum severity of disease was less than 100%. Plant height showed a larger range with the average shortest 55 cm and the highest 148 cm while DTA was observed in a range of 13 days in 2017 and 20 days in 2015 (Table 1 and Supplementary Figure 2). For both INC and SEV, moderate to high broad sense heritability was observed with the two sites under artificial inoculation showing lower heritability than the natural infection site (Table 1). HT showed the highest heritability, while DTA had the lowest heritability (Table 1). For FHB INC and SEV, moderate to high correlations were observed in all tested environments (years and sites). Generally, both HT and DTA had very significant negative correlations with INC and SEV (Figure 3). Analysis of variance (ANOVA) revealed that genotypic effects were significant for all phenotypic traits (*P* < 0.001, Supplementary Table 2).

**TABLE 1:** Mean, range and heritability of the durum association mapping panel (AM) for FHB incidence, FHB severity, plant height (cm), and days to anthesis (DTA) for the individual trial in Morden, Brandon, and Indian Head across the 2015–2017 trial series, and across sites between Modern and Brandon.

Sites	Traits	Year	Mean	Max	Min	*H*^2^
**Morden**	FHB incidence	2015	83.0	100	15	0.82
		2016	90.3	100	10	
		2017	89.3	100	0	
		Overall	87.5	100	0	
	FHB severity	2015	63.9	100	10	0.86
		2016	66.8	100	10	
		2017	67.3	100	0	
		Overall	66.0	100	0	
	Plant height	2015	94.9	135	55	0.94
		2016	99.6	148	62	
		2017	92.4	136	55	
		overall	96.6	148	55	
	Day to anthesis	2015	59.1	72	52	0.56
		2016	62.7	73	54	
		2017	67.6	76	63	
		Overall	63.3	76	52	
**Brandon**	FHB incidence	2015	84.8	100	0	0.86
		2016	85.0	100	0	
		2017	76.2	100	0	
		Overall	82.0	100	0	
	FHB severity	2015	70.7	100	0	0.77
		2016	42.3	100	0	
		2017	39.2	100	0	
		Overall	50.7	100	0	
**Morden and Brandon**	FHB incidence		84.0	100	0	0.72
	FHB severity		58.4	100	0	0.67
**Indian Head**	FHB incidence	2015	70.6	100	0	0.60
		2016	33.1	100	0	
		Overall	51.6	100	0	
	FHB severity	2015	9.5	40	0	0.59
		2016	22.7	60	0	
		Overall	16.1	60	0	

*Maximum (Max) and minimum (Min) values observed for traits, broad sense heritability coefficient (*H*^2^).*

### GWAS Analysis of FHB Resistance, HT and DTA

**FIGURE 4:**
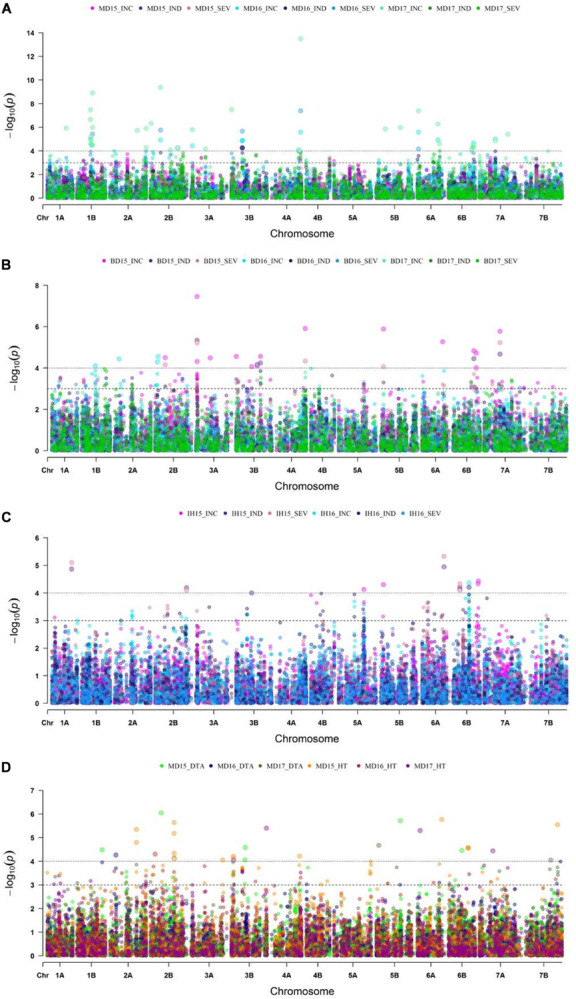
Manhattan plots displaying genome wide marker-trait association analysis for FHB incidence (INC), index (IND) and severity (SEV) at **(A)** Morden, MB from the years 2015 to 2017; **(B)** Brandon, MB for years 2015 to 2017; **(C)** Indian Head, SK from 2015 to 2016 (with natural infection); and for **(D)** plant height (HT) and day to anthesis (DTA) at Morden, MB for 2015 to 2017 trials.

**FIGURE 5:**
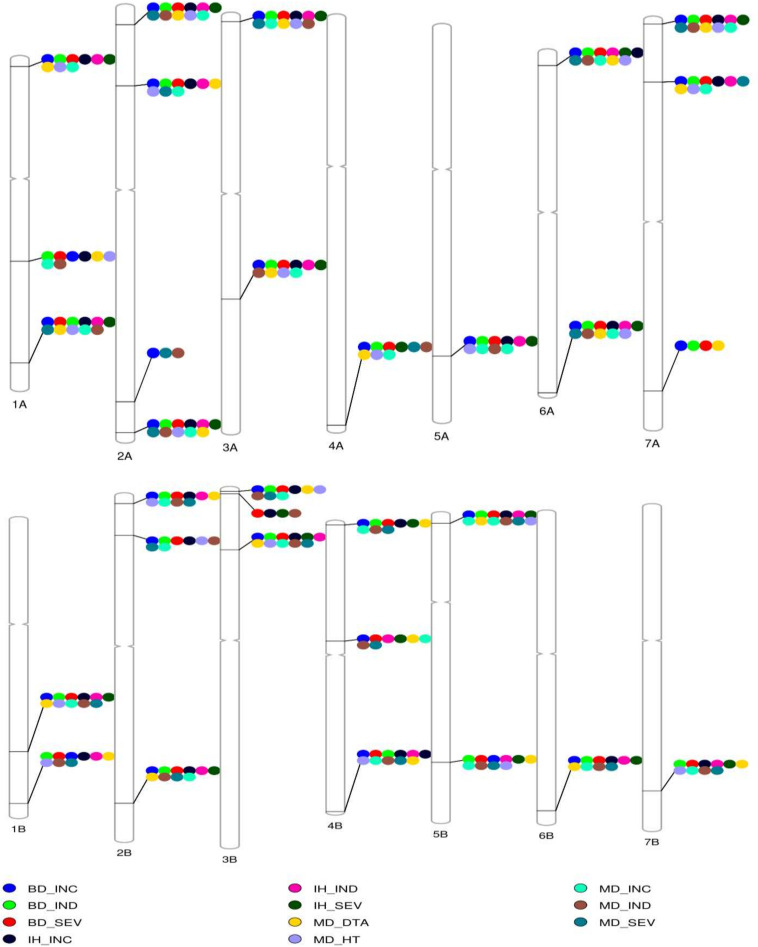
The reference genotype–phenotype map. A reference genotype–phenotype map with the most significant trait-associated markers in each chromosome aligned to the reference sequence of common wheat (International Wheat Genome Sequencing Consortium [IWGSC], 2018). MB, Morden, MB; BD, Brandon, MB; IH, Indian Head, SK. FHB incidence (INC), severity (SEV), index (IND), plant height (HT), and days to anthesis (DTA).

With GWAS analysis, 31 genomic regions were significantly associated with FHB resistance traits (Figures 4, 5). The quantile-quantile (QQ) plots (Supplementary Figure 3) showed that, for the majority of traits, an appropriate model was fitted for the GWAS test. The GWAS results were summarized in Table 2 and Supplementary Table 3. SNPs located within the same region were grouped into QTL, and Table 2 shows the QTL names and physical location of the associated SNPs based on their location on the IWGSC Chinese Spring (CS) reference 1.0 (CS Ref 1.0; International Wheat Genome Sequencing Consortium [IWGSC], 2018). For each significant QTL, the lowest –log10 (*p*-value) is shown for each environment and trait tested whenever the *p*-value is less than *p* = 0.05. As shown in Table 2, there was significant variation in detection of QTL across all of the environments, and more detection of INC than SEV across the environments. The majority of the FHB resistance QTL colocalized with DTA and/or HT.

**TABLE 2:** Quantitative trait loci names, physical positions, associated traits, explained phenotypic variance and significance of association with Fusarium head blight incidence (INC), index (IND), severity (SEV), days to anthesis (DTA), and plant height (HT) identified from durum association mapping panel across environments.

		DTA			HT			FHB incidence								FHB index								FHB severity								
*QTL name*	Physical position (Mb)	MD2015	MD2016	MD2017	MD2015	MD2016	MD2017	BD2015	BD2016	BD2017	IH2015	IH2016	MD2015	MD2016	MD2017	BD2015	BD2016	BD2017	IH2015	IH2016	MD2015	MD2016	MD2017	BD2015	BD2016	BD2017	IH2015	IH2016	MD2015	MD2016	MD2017	Max R^2^
***1A.1***	13.4–20.9	0.3	1.2	0.3	0.8	0.2	0.5	0.9	1.8	1.8	2.4	0.0	0.0	**3.2**	2.9	0.8	1.5	0.2	1.0	0.8	0.3	2.7	0.8	1.0	2.3	0.1	1.4	0.2	0.4	**3.4**	0.9	8.1
***1A.2***	366	0.0	0.4	0.3	0.2	0.3	1.9	**3.6**	0.9	2.1	2.1	0.5	0.7	2.1	1.5	**3.4**	1.9	2.6	0.5	0.4	1.4	1.8	1.7	**3.2**	1.2	2.5	0.5	0.2	1.2	1.9	2.0	8.6
***1A.3***	503–580	2.3	1.9	2.6	1.3	1.8	0.4	0.2	**3.2**	1.4	0.2	1.7	2.2	1.6	1.1	0.8	3.0	1.8	0.4	0.6	1.6	1.9	1.8	0.7	**3.4**	1.5	0.2	0.6	0.4	1.7	1.4	7.8
***1B.1***	544–581	1.9	0.2	**4.9**	0.0	1.6	1.4	2.7	**4.3**	**3.2**	1.6	1.7	0.5	**4.4**	**7.9**	1.6	1.9	0.3	1.5	1.8	0.2	3.0	2.1	2.1	2.6	1.0	1.4	0.2	0.0	**5.2**	1.5	20.3
***1B.2***	662–668	1.0	0.3	0.0	2.0	1.6	0.6	0.6	1.9	1.8	0.4	1.2	0.3	1.0	1.5	0.8	**3.6**	**3.4**	1.2	1.2	2.1	1.3	2.2	0.9	1.8	**4.0**	1.1	0.2	2.4	1.0	1.8	8.0
***2A.1***	30–31	2.6	**3.4**	0.6	3.0	2.0	2.6	2.8	**3.5**	1.4	2.0	2.6	**3.3**	**3.7**	1.8	2.6	2.5	1.2	0.5	1.8	1.7	**3.3**	2.9	1.9	**3.7**	2.2	0.4	1.1	0.2	1.7	2.2	9.0
***2A.2***	138–142	1.9	2.3	3.0	0.7	0.4	1.0	2.2	2.3	**3.1**	2.6	**3.4**	**3.9**	1.9	0.8	**3.6**	2.1	**3.3**	2.0	1.6	**3.4**	1.7	2.5	**3.0**	1.9	2.3	0.8	0.6	1.6	0.7	2.2	10.1
***2A.3***	713–717	0.2	2.4	**3.3**	**4.7**	1.8	0.6	**3.9**	2.3	1.5	1.8	0.3	0.3	0.1	1.9	1.6	0.6	0.3	0.1	0.1	0.9	0.7	1.1	2.6	0.7	0.6	0.3	0.7	0.7	1.2	1.7	9.5
***2A.4***	762–769	1.8	2.2	0.7	1.5	**4.0**	2.1	2.7	2.4	1.8	2.6	2.6	0.3	**3.4**	**5.2**	2.3	1.7	2.5	1.3	2.4	0.6	**3.5**	**3.3**	2.6	2.1	2.0	1.3	0.7	0.2	**3.8**	**3.2**	12.9
***2B.1***	8.6–22	3.0	2.6	**4.3**	**3.2**	**3.2**	1.7	1.8	**4.3**	2.0	0.3	1.9	2.8	**3.6**	**6.0**	2.0	**3.7**	1.7	0.3	1.8	0.3	2.3	2.6	2.7	2.2	0.0	1.1	1.0	0.2	**3.5**	2.6	15.1
***2B.2***	92–102	0.0	0.8	0.8	0.7	1.7	1.6	**3.3**	2.1	2.9	0.7	0.9	0.2	**4.7**	**8.1**	2.2	1.2	0.4	0.6	1.0	0.7	**3.5**	1.8	**3.2**	2.2	1.0	1.2	0.3	0.7	**5.5**	1.5	20.8
***2B.3***	717–781	0.4	1.5	**3.3**	0.4	0.9	1.8	**3.1**	3.0	2.0	1.8	1.8	1.4	1.6	2.6	2.5	1.6	1.6	1.6	1.8	0.9	1.9	2.8	**3.1**	1.8	1.9	1.0	1.7	0.4	1.6	**3.3**	7.9
***3A.1***	9.6–13	1.6	0.3	2.9	0.6	1.7	2.2	**6.2**	1.5	1.7	2.2	1.4	0.5	**4.6**	**5.4**	**4.6**	1.6	**3.3**	1.5	1.7	0.4	2.9	2.0	**4.6**	2.1	2.5	1.7	1.3	2.0	**3.2**	2.4	15.4
***3A.2***	512–556	0.3	0.3	0.6	**3.8**	**4.5**	2.6	**4.1**	2.0	1.6	1.0	2.8	0.5	2.5	**4.8**	2.3	1.4	1.4	0.5	**3.7**	0.6	1.5	**4.0**	2.6	0.8	1.3	0.6	1.8	0.2	2.8	**3.9**	11.9
***3B.1***	3.7	1.4	0.8	**4.0**	0.9	1.4	2.3	**4.1**	**3.2**	2.9	2.5	0.8	0.1	**3.1**	**8.1**	**3.4**	1.1	1.8	0.8	0.5	0.2	**3.2**	2.7	**3.5**	1.1	1.4	0.6	0.5	0.3	**4.5**	2.6	20.8
***3B.2***	9.8	1.0	0.8	1.2	0.7	0.4	0.8	1.6	0.4	1.1	**3.2**	1.4	0.2	0.6	0.2	1.7	1.6	1.5	0.8	1.5	1.1	0.6	0.6	1.8	0.8	0.5	0.0	0.2	2.0	0.2	0.6	7.7
***3B.3***	148–233	0.0	1.4	2.2	**4.5**	**3.5**	2.9	3.0	2.3	1.7	2.1	2.5	0.1	**3.8**	1.8	2.5	2.9	1.5	1.4	**3.2**	0.1	**3.7**	2.4	2.6	2.6	0.8	1.8	2.3	0.0	2.5	2.3	9.2
***4A.1***	664–737	1.7	0.9	**4.9**	**3.5**	2.8	**3.3**	**4.5**	**3.4**	**3.8**	0.6	0.6	0.7	**5.5**	**11.8**	2.1	0.8	1.5	0.6	0.1	0.8	**3.8**	3.0	**3.4**	2.2	1.6	1.6	1.6	0.4	**7.2**	**3.3**	31.8
***4B.1***	3.8	0.9	1.5	0.4	0.7	1.0	1.3	2.5	**4.0**	0.6	**4.3**	0.3	2.0	**3.2**	2.0	2.4	1.9	0.3	2.3	0.3	1.0	2.8	**3.3**	1.7	1.5	0.3	1.3	0.5	0.6	1.8	**3.4**	10.5
***4B.2***	197–347	0.4	1.5	0.3	0.1	0.6	0.3	1.3	1.9	**3.7**	1.2	0.7	0.2	1.8	1.9	0.5	0.9	2.2	1.4	0.7	0.1	1.8	2.6	0.6	1.0	1.6	1.3	2.0	0.1	1.1	2.7	7.3
***4B.3***	673	0.0	0.4	2.0	2.7	0.6	1.8	1.5	2.5	2.3	2.2	**3.1**	**3.1**	2.0	3.0	2.3	1.8	2.4	2.2	**3.0**	**3.1**	2.6	**4.2**	2.4	2.5	2.7	1.4	**3.2**	2.2	1.8	**3.8**	8.8
***5A.1***	585–591	0.4	0.6	0.4	1.7	1.1	0.5	**3.8**	1.2	2.0	**4.3**	2.6	2.7	0.5	0.3	**3.5**	1.9	1.5	1.5	**3.5**	1.7	1.1	0.3	**3.6**	2.2	1.1	0.9	2.2	0.6	0.8	0.2	8.8
***5B.1***	19.5	0.2	1.5	**4.6**	1.0	0.9	2.0	**6.6**	2.8	2.1	**5.2**	0.8	1.0	1.1	2.6	**4.2**	1.0	0.6	2.5	0.9	1.0	1.3	1.4	**5.0**	0.7	0.1	1.4	1.6	0.4	1.3	1.5	14.5
***5B.2***	577–691	1.5	2.1	1.3	0.1	1.4	0.7	1.2	2.8	1.7	1.3	1.2	**3.1**	2.9	**3.7**	1.5	2.1	1.4	0.1	2.1	2.5	2.5	1.7	0.4	1.1	0.0	0.1	1.8	1.5	2.8	1.5	9.6
***6A.1***	12–23	0.7	1.3	1.4	0.3	1.5	1.3	2.7	2.4	**3.2**	0.6	2.4	0.6	**5.8**	**6.3**	2.4	2.6	2.4	0.2	1.4	0.5	**3.8**	**3.4**	2.1	**3.3**	2.9	0.5	1.4	2.2	**4.0**	**3.4**	15.7
***6A.2***	601–694	1.7	1.8	1.9	0.1	0.5	2.5	**4.1**	**3.5**	**1.6**	1.7	0.4	0.6	**5.2**	**6.4**	2.2	0.9	2.2	**3.8**	1.0	0.8	2.6	**3.7**	2.0	1.5	2.6	**3.3**	1.7	1.5	1.9	**4.2**	16.0
***6B.1***	585–707	1.8	1.8	1.9	0.5	0.2	0.4	**4.0**	3.0	1.4	2.2	0.0	0.3	**5.0**	**4.1**	2.5	1.0	0.2	2.5	0.2	0.1	**3.5**	1.1	2.5	0.5	0.5	1.1	0.1	0.1	**4.0**	0.9	12.2
***7A.1***	7.5–12	2.0	0.5	0.8	1.8	0.9	**3.1**	1.4	2.0	**3.7**	2.7	1.4	1.4	2.7	**5.5**	**3.6**	1.6	2.1	0.6	1.5	1.1	1.9	2.0	**4.0**	1.4	0.2	1.8	1.0	0.1	2.7	1.9	11.1
***7A.2***	102–113	1.5	2.1	2.6	1.1	0.3	1.4	**5.1**	**3.3**	**3.7**	2.7	0.7	0.5	**5.4**	**5.2**	**4.7**	1.6	0.5	0.9	0.5	0.0	**4.5**	2.8	**5.0**	0.5	0.0	0.3	0.4	0.0	**3.8**	**3.4**	13.5
***7A.3***	671	0.2	0.3	0.2	0.5	0.3	0.2	**3.9**	0.6	1.8	2.7	0.7	0.2	2.1	1.7	**3.0**	1.9	1.8	0.8	1.1	0.4	2.3	0.4	**3.0**	1.9	2.5	0.3	0.8	0.5	2.0	0.4	9.6
***7B.1***	610–658	1.7	0.1	**6.0**	0.4	1.6	1.1	**5.2**	**4.0**	2.5	**3.6**	0.9	1.5	1.9	**4.7**	**4.0**	2.0	2.6	1.4	1.0	1.7	1.9	1.8	**4.0**	0.7	2.9	2.1	0.1	1.1	1.7	1.6	12.9

*The highest –log10 (*p*-value) of the markers from the QTL is given across all traits measured from the field trials performed in Brandon (BD), Morden (MD), and Indian Head (IH) in the years 2015, 2016 and 2017. –log10 (*p*) > 3 are in bold, values above the stringent Bonferroni significance threshold are underlined, and values below –log10 (*p*) = 1.3 are not shown. Physical position (Mb): physical location of SNP markers in the QTL from Chinese Spring assembly (International Wheat Genome Sequencing Consortium [IWGSC], 2018). Max *R*^2^: highest value for explained phenotypic variation for the marker across the traits and environments tested, expressed as%.*

A major QTL, *1B.1*, was found between 544 and 580 Mb on 1B (Figure 5 and Table 2). It was significant for INC, SEV and IND, and explained as much as 20% of the phenotypic variation (Table 2 and Supplementary Table 3). The QTL *1A.3* was located in the syntenic region of *1B.1*, between 503 and 580 Mb (Figure 5 and Table 2), and it was also significant for IND and INC though present in fewer environments and with lower significance than *1B.1* (Table 2). *1B.1* colocated with significant HT and DTA QTL, while 1A.3 was significant for DTA.

Another major QTL was at 30–31 Mb on 2AS, termed *2A.1* (Figure 5 and Table 2). This QTL was significant for INC, IND and SEV, as well as being associated with HT and DTA (Figures 4, 5, Tables 2, and Supplementary Table 3). It was one of the more stable QTL detected, being present for INC in all environments. Another significant QTL, *2B.1*, was located between 8.6 and 22 Mb and was associated with all tested traits and explained up to 15% phenotypic variation (Table 2 and Supplementary Table 3). QTL 2A.2 was also stable, and detected for INC in seven, IND in eight and SEV in five environments (Supplementary Table 3). It was located from 138 to 142 Mb, and was consistently associated with DTA (Table 2 and Supplementary Table 3). QTL *2A.2* explained up to 10% of phenotypic variation (Table 2). On group 5, the QTL *5A.1* in the region between 585 and 591 Mb of 5A had a relatively stable effect for INC in both Brandon, MB, and Indian Head, SK (Figures 4, 5 and Table 2). It was detected at a low level for HT in one environment. 5B.2 was located from 577 to 691 Mb on 5BL (Table 2). It explained up to 9.6% of phenotypic variation, and was most stable for INC in Brandon and Morden. This QTL was also associated with DTA, and minor effects were observed on IND and SEV, including at Indian Head (Figure 4 and Table 2).

**FIGURE 6:**
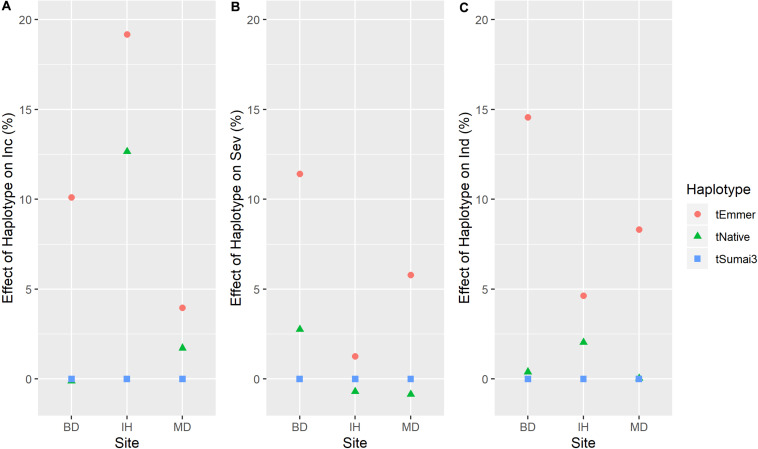
Haplotype effects (mean values across years in each site) of *Fhb1* (3BS.2) QTL on **(A)** FHB incidence (Inc); **(B)** FHB severity (Sev); and **(C)** FHB index (Ind). Three types of haplotype were identified and defined as tEmmer, tNative and tSumai3. Site: BD, Brandon, MB; IH, Indian Head, SK; and MD, Morden, MB. *Y*-axis, effects of haplotype on disease susceptibility, larger number indicates that haplotype increases disease susceptibility.

Three QTL were identified on chromosome 3B (Figure 5). The *3B.1* QTL was located around 3.7 Mb. It was identified in significant levels for INC, IND and SEV, and explained as much as 20.8% of phenotypic variation (Table 2). The QTL also affected HT with a very large effect on DTA. A stable QTL, designated *3B.3*, was located on chromosome 3B at 141–233 Mb (Table 2). This QTL affected up to 9% of phenotypic variation, and also conferred a very stable effect on HT and smaller effect on DTA (Table 2). The third 3B QTL, *3B.2*, was located around 9.8 Mb (Table 2), approximately 1 Mb from *Fhb1* in common wheat (Rawat et al., 2016; Li et al., 2019; Su et al., 2019). It had no observable effect on HT or DTA, but also had a quite minor effect, explaining at most 7.7% of phenotypic variation (Table 2). Though this QTL was less stable, because of the location of *3B.2* in the region of *Fhb1* and because of the importance of this gene to FHB resistance in common wheat, we chose to further characterize the QTL in the durum AM panel. Pedigree information and genotypes of *3B.2* identified three different haplotypes for the significant marker, BS00079522_51, which were defined as tSumai3, tNative and tEmmer types (Supplementary Table 4). The tSumai 3 haplotype was derived from the introgression of *Fhb1* from Sumai 3 into durum wheat (Supplementary Table 4). All Canadian cultivars shared the tNative haplotype, and the tEmmer haplotype was found in durum wheat introgressed from Td161 and a few durum wheat experimental lines from Austria (Supplementary Table 4). Allele effect analysis identified that the tEmmer type of *3B.2* conferred an effect that increased disease susceptibility (Figure 6).

There were a small number of QTL that did not co-locate with DTA or HT QTL. These include *1A.1*, *1A.2*, *6A.1*, and *7A.3*. The QTL *1A.1*, located near the distal end of the short arm of chromosome 1A within a region from 13 to 20 Mb, was only significant for INC. Also on 1A was *1A.2*, which mapped to 366 Mb on chromosome 1AL. It was detected in seven of the eight different environments, though not consistently across INC, IND and SEV, and a minor association with HT was also identified in one environment at this locus (Figure 4, Table 2, and Supplementary Table 3). *6A.1* was positioned at 12–23 Mb on 6AS. It had a significant effect on FHB, explaining up to 16% of phenotypic variation. This QTL also had a very minor effect for both HT and DTA with each only observed in a single environment. The *7A.3* QTL located to the distal region of 7A, around 671 Mb, had an effect on INC, SEV and IND, with no QTL for height or DTA found in this region (Figure 4 and Table 2). This QTL was detected only in Brandon and Morden field sites, and explained up to 9.6% phenotypic variation (Table 2).

## Discussion

### Phenotypic Data Analysis

The moderate to high heritability observed for FHB resistant traits in multiple environments in the durum AM panel indicated a large part of the phenotypic variation was contributed by genetic variation. The positive correlation between plant height and days to anthesis indicated that the genetic control of plant height and flowering time was partially shared (Table 1; Langer et al., 2014). The high proportion of disease susceptibility we observed in the field tests supports literature emphasizing the limited tetraploid wheat resources with a high level of FHB resistance (Oliver et al., 2008). The observed significantly negative correlations between FHB resistance and plant height and days to anthesis also agreed with previous findings summarized by Prat et al. (2014) and Steiner et al. (2017). Because the significant negative correlations between both DTA and HT and FHB traits ranged from −0.24^∗∗∗^ to −0.60^∗∗∗^ and −0.18^∗^ to −0.42^∗∗∗^, respectively, there is considerable scope to shift this negative relationships (i.e., to have DTA more consistently around −0.24 and the correlation with HT toward −0.18). By adopting strategies to stratify experimental genotypes into groups by both days to anthesis and plant height, it may be possible to recombine earlier to flower and shorter plants with reduced FHB symptoms. The correlation will not be broken but it can be shifted so that earlier maturing and shorter genotypes can be recombined with reduced FHB symptoms. Using this strategy, the negative relationship between plant height and FHB traits has been shifted by recombining semi-dwarf stature with a moderate level of resistance in hexaploid wheat cultivars such as Carberry (DePauw et al., 2011) and AAC Brandon (Cuthbert et al., 2017), both of which became widely adopted by producers. Adopting this strategy in durum wheat genetic enhancement could prove equally effective.

### Genetic Architecture of FHB Resistance in the Durum AM Panel and Its Association With Flower Time and Plant Height

Compared to common wheat, durum wheat has limited genetic variation, and less effort has been committed to improve durum resistance to FHB (Buerstmayr et al., 2009, 2019; Prat et al., 2014, 2017). Within the current study, we identified a large number of QTL associated with FHB resistance with GWAS analysis from multiple environments and sources, broadening the resistance gene pool in durum wheat. The minor effect of these multiple QTL reinforces what is already known about the polygenic nature of FHB resistance, but also reveals the necessity of combining genes from multiple sources (Buerstmayr et al., 2009, 2019; Liu et al., 2009).

The major and most consistent FHB QTL found in previous studies is the hexaploid wheat Sumai 3 derived *Fhb1*, located on 3BS around 7.6–13.9 Mb (Anderson et al., 2001; Liu et al., 2006).

Introgression of *Fhb1* into durum wheat has been challenging, with one possible reason being the unstable expression in a durum genetic background (Zhao et al., 2018). Recently, Prat et al. (2017) successfully introgressed *Fhb1* into durum wheat, and some of those introgression lines are part of this AM panel. A QTL was found in the same region as *Fhb1* in this study, designated *3B.2.* This QTL was detected in limited environments with a minor effect. QTL *3B.2* had three distinct haplotypes (Supplementary Table 4), and compared to haplotypes of Sumai 3 (tSumai 3) and Canadian cultivars (tNative), the haplotype from the experimental lines derived from emmer wheat Td161 (tEmmer) conferred disease susceptibility (Supplementary Table 4). This finding confirms previous findings that the *Fhb1* region from Td161 contributed to disease susceptibility when compared to the susceptible durum wheat Floradur (Buerstmayr et al., 2012). The resistance haplotype found in the GWAS study by Steiner et al. (2019b) corresponds to the tNative haplotype presented in this study. The tNative haplotype is the only haplotype found in the Canadian and American cultivars presented in both studies, while both the tNative and tEmmer haplotypes exist in durum wheat from Austria, CIMMYT, ICARDA, Italy and Morocco (Steiner et al., 2019b). Altogether, these findings indicate that one of the two non-Sumai 3 *Fhb1* region haplotypes found in tetraploid wheat contributed to disease susceptibility when compared to the other. Further characterizing the region with additional markers is needed to help resolve the source of the alleles and further understand the effects of the three haplotypes identified in this study.

Two additional 3B QTL were found significantly associated with all of the traits, *3B.1* in the telomeric region of 3BS, and *3B.3* in the centromeric region of the short arm (3BSc). Recently, Wu et al. (2019) reported a QTL positioned at 2.0 Mb on the reference sequence from elite Chinese common wheat germplasm, almost the same region as the *3B.1* identified in this durum AM panel study. The *3B.3* QTL was one of the most stable QTL identified, with a larger effect on FHB resistance than other QTL in this AM panel. Notably, the resistant 3BSc haplotypes were identified in the durum wheat lines that also had *Fhb1* introgressed from Sumai 3 by Prat et al. (2017). The location of *3B.3* corresponds to the 3BSc region QTL previously reported as important to FHB resistance, particularly in Canadian elite germplasm, where 3BSc conferred a larger effect than *Fhb1* (McCartney et al., 2007). Also in agreement with findings from McCartney et al. (2007), the 3BSc QTL conferred a large effect on both plant height and DTA in elite Canadian wheat. Further research is needed to explore effects of *Fhb1*, *3B.1* and *3BSc* in durum wheat.

### QTL With No or Weak Association With Flowering Time and Height

The common association between plant height, flowering time and FHB resistance was illustrated in this study. Of the 31 FHB QTL regions identified, all but five also had strong associations with plant height and/or flowering time. The relatively small effects of these QTL compared to other QTLs detected in this study may be related to the strong influence of flowering time on FHB resistance, potentially overinflating the effects of the QTL for FHB resistance due to the timing of flowering. Due to the progression of the FHB symptoms over time, the correlation between days to anthesis and disease development are confounded by the length of time for disease development. Due to cost constraints, disease rating was not evaluated over a time course to control for this effect, and thus we cannot exclude the observed correlation between FHB resistance and DTA may be caused by these confounding effects.

Fusarium head blight resistance QTL that are not associated with height or flowering time are much more appealing targets, as the negative influence of taller plants and complicated relationship with flowering time can be avoided. The targeted breeding of these QTL for resistance that do not carry extra undesirable traits will have the most likely success. The most favorable of these QTL may be *3B.2*, but the QTL *1A.1*, *1A.2*, *6A.1*, and *7A.3* with no association or weak association with DTA and HT are also desirable candidates. The *1A.1* QTL was located in the same region as the major QTL previously reported on the distal part of 1AS (summarized by Buerstmayr et al., 2009; Liu et al., 2009; Venske et al., 2019). Jiang et al. (2007a, b) located an FHB SEV QTL from the Chinese wheat line CJ9306 to position 27.2 Mb, and GWAS by Zhu et al. (2020) similarly identified an FHB QTL for IND from Chinese elite germplasm in the same region. A recent study by Sari et al. (2018) of *T. carthlicum* cv. Blackbird identified an important FHB QTL for INC, SEV and IND in the region of 1AS that agrees well with the *1A.1*. The *1A.2* QTL colocalized with a QTL positioned at around 350 Mb for FHB severity and DON identified in Chinese elite germplasm (Wu et al., 2019) and for FHB resistance based on point inoculation in CIMMYT line C615 (Yi et al., 2018). In our study, we found this QTL was also associated with FHB incidence, index and severity. Within the AM panel of our study, although the resistance allele of *1A.1* was not found in Canadian cultivars, the *1A.2* occurred in several current Canadian cultivars with improved FHB resistance, including CDC Precision (Pozniak and Clarke, 2017b) and Brigade (Clarke et al., 2009; Supplementary Table 3).

The *6A.1* QTL’s large effect on FHB resistance makes it appealing despite a small undesirable influence on DTA and HT. No major QTL clusters have been reported in a similar region as *6A.1*, though Yi et al. (2018) reported a minor QTL in this region detected from a susceptible wheat line in one environment, and Lu et al. (2013) identified a minor QTL in the proximal 6A region for both FHB resistance and plant height. Because the *6A.1* resistance haplotype is present in a large number of Canadian durum wheat cultivars, including Brigade (Clarke et al., 2009), Transcend (Singh et al., 2012), CDC Credence (Sari et al., 2018) and CDC Precision (Pozniak and Clarke, 2017b; Supplementary Table 3), it should be possible for Canadian breeding programs to build on this resistance, though the effect of the QTL in Canadian elite durum cultivars remains to be validated.

The *7A.3* QTL, located at 671 Mb, with its relatively large effects on all FHB resistant traits without being associated with plant height or flowering time also make it another good target for breeding FHB resistance. Previous research identified a major QTL for type II resistance based on point inoculation in the vicinity of *7A.3* through the physical mapping of the SSRs *gwm276* and *gwm262* to positions of 642.9 and 681.4 Mb (Semagn et al., 2007; Buerstmayr et al., 2009). Wu et al. (2019) also reported a QTL affecting DON accumulation in the same region of elite Chinese germplasm, while Sari et al. (2018) reported QTL for SEV and IND in the same region from the durum wheat inbred line DT696.

From the durum AM panel in our study, *2A.2* located in the same region as a native durum FHB resistance QTL in previous research in cultivars Ben by Zhang et al. (2014) and Joppa by Zhao et al. (2018). In addition, the QTL *2A.2* was also found consistently associated with DTA, suggesting it plays a role in controlling flowering. In this durum AM panel, the resistance haplotype of *2A.2* was found in DT696 (Sari et al., 2018), an adapted source of FHB resistance in durum wheat, as well as several Canadian cultivars with improved FHB resistance derived from this line, including Brigade (Clarke et al., 2009), Transcend (Singh et al., 2012) CDC Credence (Sari et al., 2018), and CDC Precision (Pozniak and Clarke, 2017b; Supplementary Table 3). Despite its association with DTA, the effectiveness of the 2A.2 in native durum cultivars from Canada and United States make it another good target to breed durum wheat with improved FHB resistance.

### QTL Co-located With Flowering Genes

The majority of the QTL identified from this AM panel were found associated with flowering time and/or plant height. As mentioned previously, the Notably, three QTL pairs, including *1A.3* and *1B.1*, *2A.1* and *2B.1*, and *5A.1* and *5B.2*, were found in syntenic regions of the A/B genome that harbor known orthologous gene pairs controlling flower time. 1A.3 was in a similar region of a major QTL found in United States winter wheat cultivar NC-Neuse (Petersen et al., 2016, 2017). The *FLOWERING LOCUS T3-A1* (*TaFT3-A1*) gene that promotes flowering was found physically mapped around 528.1 Mb of 1A in CS Ref 1.0 (Zikhali et al., 2017; International Wheat Genome Sequencing Consortium [IWGSC], 2018), which is close to the region of *1A.3*. The major QTL *1B.1* located to the region coinciding with a QTL of FHB resistance from the European winter wheat Arina (Semagn et al., 2007; Buerstmayr et al., 2009; Liu et al., 2009), as well as loci controlling DTA identified in the recent durum wheat GWAS by Steiner et al. (2019b). This QTL conferred a stable and large effect for INC, SEV, HT and DTA. Recently, the photoperiod gene *FLOWERING LOCUS T3-B1* (*TaFT3-B1*) that promotes flowering time, was physically identified at position 581 Mb of 1B (Zikhali et al., 2017), the same region as *1B.1*. The *1B.1* and *1A.3* QTL occur in syntenic region of the genome, indicating the orthologous gene pair, *TaFT3-B1* and *TaFT3-A1*, as candidate genes underling the QTL effect in these regions.

The *2A.1* QTL conferred main effects for INC, IND, DTA and HT, physically positioned to around 27–31 Mb on chromosome 2A. This location is very near to the photoperiod gene *Ppd1A*, which has an important role in controlling flowering time and height, indicating *2A.1* as candidate gene controlling the QTL. Giancaspro et al. (2016) found a similar QTL positioned at 10 Mb on 2AS for FHB resistance in durum wheat, derived from the introgression of FHB resistance from Sumai 3, but with no report on its association with plant height. Gadaleta et al. (2019) identified a wall-associated receptor-like kinase (WAK2) in this region as the candidate gene for FHB resistance. Our study found a 2B QTL, designated *2B.1* that colocalizes with *Ppd1B* located in a syntenic region of *2A.1*. This QTL contributed to INC, SEV, IND, DTA and HT. Thus, our findings support the *Ppd* loci on 2AS and 2BS as candidate genes responsible for the observed effects, although further studies with well stratified plant height and FHB rating DTA are required in order to explore the factors underlying these QTL.

Both the QTL *5A.1* on 5AL and *5B.2* on 5BL occur in syntenic regions that harbor orthologs of the well-known vernalization genes *VRNA1* (at 585.1 Mb) and *VRNB1* (at 613.0 Mb). *5A.1* and *5B.2* both conferred a stable effect for INC and IND, and while *5B.2* also had a large effect of on DTA, *5A.1* had no effect on DTA and only a minor effect on HT in one environment. Sari et al. (2018) reported a major FHB resistance QTL from the Canadian durum wheat line DT696 in the same region as *5A.1*, also finding no DTA or HT QTL in this region. Xu et al. (2020) found QTL located in the same regions as *5A.1* and *5B.2* in common wheat that controlled anther extrusion, heading time and FHB resistance. There is potential that these vernalization genes are responsible for the FHB resistance coming from these regions, and that the *VRNA1* gene has just a minor effect on flowering time in durum wheat. The resistance haplotype of *5A.1* was found in Canadian durum cultivars including Brigade (Clarke et al., 2009) and CDC Alloy (Pozniak and Clarke, 2017a; Supplementary Table 3). Because of the presence of the resistant haplotype in current durum cultivars, and the minor effect on flowering time, we believe the *VRNA1* region QTL from this study and Sari et al. (2018) is a good target to improve FHB resistance in durum wheat. However, there is still need for further research to explore the mechanism of colocalization between the vernalization genes and FHB resistance and their effect on flowering in durum.

## Conclusion

With genome wide association analysis we identified 31 QTL for FHB resistance. This confirms the quantitative nature and polygenic control of the FHB resistance and also signifies that this durum AM panel contains a large amount of genetic variation for FHB resistance loci. These QTL capture a large amount of the major QTL reported for hexaploid and tetraploid wheat which should facilitate improving FHB resistance in durum wheat. Five QTL found primarily for FHB resistance, including *1A.1*, *1A.2*, *5A.1*, *6A.1*, and *7A.3*, could be used as initial targets to improve resistance in durum wheat without detrimental effects. Although *2A.2* is associated with DTA, the resistant haplotype exists in several Canadian and United States cultivars with improved FHB resistance, and we think that due to its adaption to durum cultivars in North America it is also a good target. The majority of these QTL identified were associated with plant height and/or flowering time, indicating that phenology, flowering and height genes formed a complex network affecting FHB resistance in durum wheat. Prior knowledge of the haplotypes of these genes in breeding materials will provide an informed approach to stack these genes and give breeders the ability to design a better strategy to use these sources to improve FHB resistance. However, more research is needed to identify the mechanism of the trait associations, and truly determine whether pleiotropic effects of same gene, linkage drag of resistant genes, and/or disease escape due to flowering time and plant height are in effect. Only by completely understanding these relationships, can a better strategy, from genetic, genomics and breeding perspectives be developed to significantly increase FHB resistance in durum wheat. Finally, considering the attributes of QTL identified in this study, including the large number of minor effects, the varied expression across environments, and the complex interaction with flowering time and height, we suggest intercrossing the multiple sources of resistance. Then the progeny should be selected using a multi-trait based, high-throughput marker assisted selection approach that incorporates resistance, flowering time and height loci, in combination with intensive phenotyping, with the genotypes grouped by days to flower and plant height, across multiple target environments, as the most promising approach to develop durum wheat with a better level of resistance.

## Data Availability Statement

The original contributions presented in the study are included in the article/Supplementary Material, further inquiries can be directed to the corresponding author/s.

## Author Contributions

YR, RD, RK, PF, and WZ conceived and designed this study. YR and RK constructed the durum association mapping (AM) population. HB, RD, RK, RC, and YR contributed to the durum germplasm development in the AM population. YR, RK, HC, and SB contributed to seed increase of this AM population and the design of field trials. MH, AB, and SK conducted field trials and disease evaluation at FHB nurseries located in Morden and Brandon, Manitoba. YR, SB, RK, RC, and HC performed field trials and disease evaluation at FHB nursery in Indian Head, Saskatchewan. KB and BP contributed to the 90K SNP genotyping and marker identification. KB, WZ, and YR analyzed the data and interpreted results. KB, WZ, RR, and YR contributed to data validation. WZ, KB, and YR wrote the original draft. KB, RK, WZ, YR, RD, HB, HC, and PF reviewed and edited the manuscript. YR was the principal investigator and supervised the project. All authors contributed to the article and approved the submitted version.

## Conflict of Interest

The authors declare that the research was conducted in the absence of any commercial or financial relationships that could be construed as a potential conflict of interest.

## Footnotes

1 http://tree.bio.ed.ac.uk/software/figtree/
