# Supplementary material for: Characterization of the Genetic Architecture for Fusarium Head Blight Resistance in Durum Wheat: The Complex Association of Resistance, Flowering Time, and Height Genes
Source: Front Plant Sci. 2020 Dec 23;11:592064. doi: 10.3389/fpls.2020.592064 (PMC7786293; doi:10.3389/fpls.2020.592064)
Supplement: Supplementary file 4 [file Data_Sheet_1.PDF]

Supplementary Material

Figure S2

Article Title: Characterization of the genetic architecture for Fusarium head blight resistance in durum wheat: the complex association of resistance, flowering time and height genes

Journal: Frontiers in Plant Science

Yuefeng Ruan, Wentao Zhang, Ron Knox, Samia Berraies, Heather Campbell, Raja Ragupathy, Kerry Boyle, Brittany Polley, Maria Antonia Henriquez, Andrew Burt, Santosh Kumar, Richard Cuthbert, Pierre R. Fobert, Hermann Buerstmayr and Ron DePauw

Name, affiliation, and email of corresponding author

Wentao Zhang

Aquatic and Crop Resources Development,

National Research Council of Canada, Saskatoon,

SK, S7N 0W9

Email: Wentao.Zhang@nrc-cnrc.gc.ca

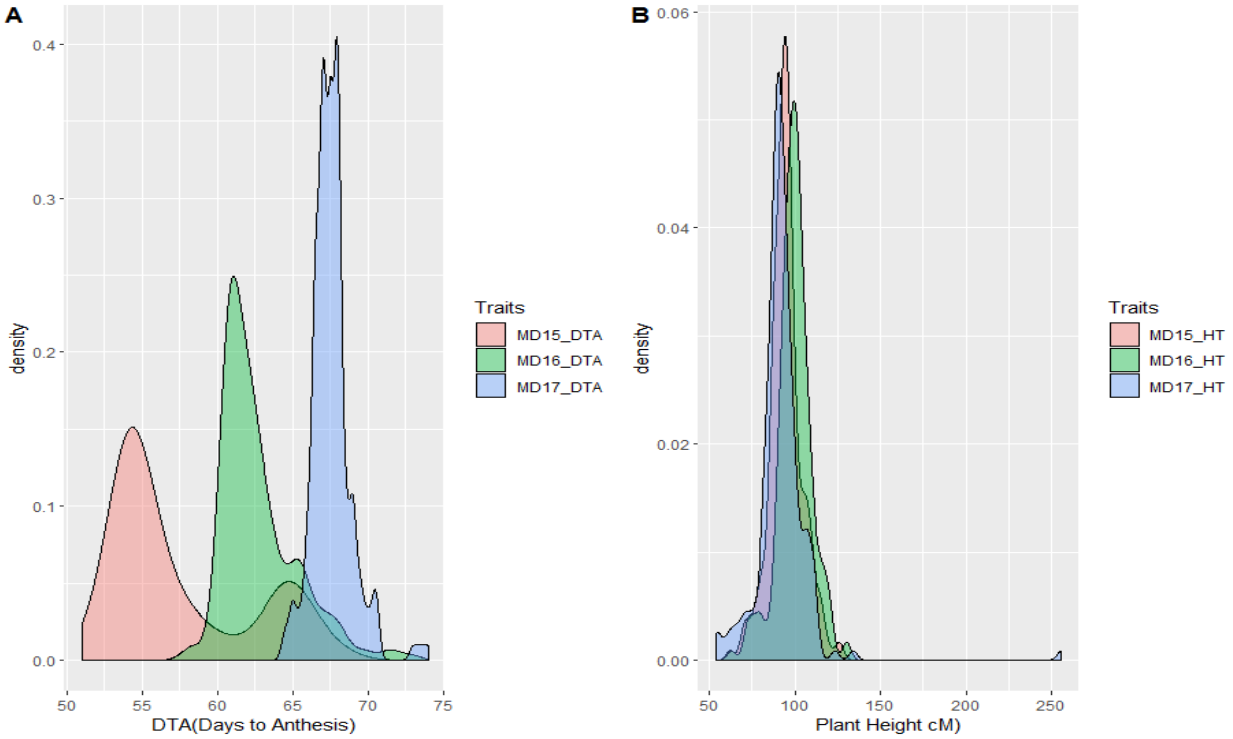

Fig. S2 Distribution of days to anthesis (A) and plant height (B) of the durum assocaition mapping panel in field trials in 2015, 2016 and 2017
