# Supplementary material for: Characterization of the Genetic Architecture for Fusarium Head Blight Resistance in Durum Wheat: The Complex Association of Resistance, Flowering Time, and Height Genes
Source: Front Plant Sci. 2020 Dec 23;11:592064. doi: 10.3389/fpls.2020.592064 (PMC7786293; doi:10.3389/fpls.2020.592064)
Supplement: Supplementary file 6 [file Data_Sheet_3.PDF]

Supplementary Material

Table S2

Article Title: Characterization of the genetic architecture for Fusarium head blight resistance in durum wheat: the complex association of resistance, flowering time and height genes

Journal: Frontiers in Plant Science

Yuefeng Ruan, Wentao Zhang, Ron Knox, Samia Berraies, Heather Campbell, Raja Ragupathy, Kerry Boyle, Brittany Polley, Maria Antonia Henriquez, Andrew Burt, Santosh Kumar, Richard Cuthbert, Pierre R. Fobert, Hermann Buerstmayr and Ron DePauw

Name, affiliation, and email of corresponding author

Wentao Zhang

Aquatic and Crop Resources Development,

National Research Council of Canada, Saskatoon,

SK, S7N 0W9

Email: Wentao.Zhang@nrc-cnrc.gc.ca

Table S2 Analysis of variance (ANOVA) of the durum association mapping panel (AM) for FHB incidence (INC), FHB severity (SEV), plant height (HT, cm), and days to anthesis (DTA) for the individual trial in Morden, Brandon, and Indian Head and across between Morden and Brandon. Variance components were partitioned as Genotype (δ^2_G), year (δ^2_{yr}), Genotype X year (δ^2_{GXyr}), location (δ^2_{loc}), Genotype X locations (δ^2_{GXloc}) and residuals (δ^2_e)

Sites	Traits	Variance components					
		δ^2_G	δ^2_{yr}	δ^2_{GXyr}	δ^2_{loc}	δ^2_{GXloc}	δ^{2e}
**Morden**	INC	108.71***	15.5	43.67			55.61
	SEV	127.92***	3.023	19.28			82.05
	HT	85.98***	13.59	0.00			34.42
	DTA	2.11***	15.96	2.98			3.85
**Brandon**	INC	384.80***	24.2	102.00			176.40
	SEV	212.08***	299.58	88.21			198.28
**Morden & Brandon**	INC	192.07***	6.13	18.92	14.37	64.35	170.29
	SEV	159.60***	60.08	0.00	115.3	9.65	255.40
**Indian Head**	INC	248.60***	263.3	680.20			488.10
	SEV	19.60***	87.16	10.20			62.61

Notes: Asterisk (***) indicates significance at $p < 0.001$
